# Perception and expectations of personal sound amplification products in Korea: A hospital-based, multi-center, cross-sectional survey

**DOI:** 10.1371/journal.pone.0269123

**Published:** 2022-05-26

**Authors:** Jae Sang Han, Yeonji Kim, Moo Kyun Park, Jae-Jun Song, Il Joon Moon, Woojoo Lee, Young Sang Cho, Jae-Hyun Seo, Yong-Ho Park

**Affiliations:** 1 Department of Otolaryngology-Head and Neck Surgery, College of Medicine, The Catholic University of Korea, Seoul, Republic of Korea; 2 Department of Otorhinolaryngology-Head and Neck Surgery, Seoul National University Hospital, Seoul National University College of Medicine, Seoul, Republic of Korea; 3 Sensory Organ Research Institute, Seoul National University Medical Research Center, Seoul, Republic of Korea; 4 Department of Otorhinolaryngology-Head and Neck Surgery, Korea University College of Medicine, Seoul, Republic of Korea; 5 Department of Otorhinolaryngology - Head and Neck Surgery, Samsung Medical Center, Sungkyunkwan University School of Medicine, Seoul, Republic of Korea; 6 Department of Public Health Sciences, Graduate School of Public Health, Seoul National University, Seoul, Republic of Korea; 7 Department of Otolaryngology-Head and Neck Surgery, College of Medicine, Chungnam National University, Daejeon, Republic of Korea; 8 Brain Research Institute, College of Medicine, Chungnam National University, Daejeon, Republic of Korea; University of Colorado School of Medicine, UNITED STATES

## Abstract

**Objectives:**

The objective of this study was to investigate current perception and expected price of personal sound amplification products (PSAPs) and to analyze influencing factors through multi-center hospital-based surveys of outpatients, caregivers, and hearing experts.

**Methods:**

A multi-center exploratory cross-sectional study was conducted in two groups of respondents with two separate surveys: 1) a perception survey of patients and caregivers who visited an otorhinolaryngology outpatient clinic in 5 general hospitals and 2) an opinion survey of hearing specialists about the expected price of PSAPs. A total of 197 outpatient visitors responded to the perception survey, and 42 hearing specialists responded to the opinion survey.

**Results:**

Overall perception score (18 questions) was 3.04 (95% CI, 3.00–3.09). When 5 categories of perception (knowledge, needs, cost, expectation, and information categories) were analyzed, cost and expectation showed the highest scores of 3.33 (95% CI, 3.21–3.44) and 3.33 (95% CI, 3.27–3.40), respectively, and needs showed the lowest score of 2.23 (95% CI, 1.97–2.49). The ≥ 60-year-old group showed significantly higher perception of PSAPs (*P* = 0.002), and the individuals with greater severity of subjective hearing loss showed significantly higher perception of PSAPs (*P* = 0.002). The expected price of PSAPs of the outpatient visitors was 933.9 USD (95% CI, 811.9–1056.0) per ear. Mean expected price of PSAPs of hearing specialists was 291.3 USD (95% CI, 238.2–344.3) per ear.

**Conclusion:**

The perception rate of PSAPs was very low, and there was a discrepancy in the expected price of PSAPs between patients/caregivers and hearing experts. Hearing specialists should make effort to improve perception of PSAPs.

## Introduction

Medical, economic and social development has led to worldwide population ageing, and it has been especially fast in Eastern and South-Eastern Asia. United Nations reported in 2019 that the number of persons aged 65 years or over was 703 million globally and 260 million in Eastern and South-Eastern Asia [1]. As the population ages, the number of persons with hearing loss is growing [2]. Hearing loss can accelerate cognitive impairment, depression, and social isolation and decrease physical activity [3]. Thus, early hearing rehabilitation is important to prevent such socio-economic burdens [4]. Hearing aids (HAs) are the first-line management to rehabilitate patients with hearing difficulties and improve quality of life [5]. Previous studies have reported that HAs can prevent cognitive decline in patients with hearing impairment, highlighting the importance of hearing rehabilitation in the elderly population [6]. However, the HA adoption rate of those with hearing impairment in developed countries is low, at 14–42% [7], and presumably is worse in developing countries [8]. Health insurance coverage for HAs in Republic of Korea is limited to bilateral profound hearing loss patients, and a nationwide study reported that prevalence of regular HA use was 12.6% [9].

There are several barriers to adopting HAs, including cost, social stigma, low motivation, and limited access to health care providers [10, 11]. To overcome these barriers, alternative hearing rehabilitation devices have been highlighted, such as personal sound amplification products (PSAPs), over-the-counter (OTC) hearing aids, and smartphone-based hearing aid applications (SHAAs) [12]. PSAPs are defined by the U.S. Food and Drug Administration (FDA) as “wearable electronic products that are intended to amplify sounds for people who are not deaf or hard of hearing.” The FDA decouples PSAPs from conventional HAs, stating that PSAPs are not targeted for hearing loss individuals. However, the market size of sound amplifiers is gradually growing with major companies such as Samsung and Bose entering the market, and the MarkeTrak 10 data reported that 9.6% of individuals with hearing difficulties adopted PSAPs, while 40.6% adopted Has [13, 14].

When compared to HAs, PSAPs lower the barrier on accessibility with their cheaper price and no requirement for consultation. The cost barrier is lower for PSAPs (approximately $20–400) than for traditional HAs ($1,000–5,000). In addition, while professional consultation is required for traditional HAs, it is not needed for PSAPs [12]. Recent studies have reported that PSAPs show similar performance to HAs for mild to moderate hearing loss [15, 16], but PSAPs have limitations for severe to profound hearing loss individuals [17]. PSAPs would be good alternatives to traditional HAs for the patients with mild-to-moderate hearing loss who are yet reluctant to adopting HAs.

Direct-to-consumer hearing devices such as PSAPs are leading changes in the hearing aid market [18]. Unlike HAs, in which consultation and fitting are necessary, PSAPs can be purchased directly over the counter. Patients could be misled if detailed information about PSAPs, including average price, when to use, and what to expect from using PSAPs, is not provided. Therefore, it is necessary for hearing professionals to recognize the perception of PSAPs among patients and caregivers and to educate them with accurate information. In order to inform the patients and caregivers, we need to understand how the patients and caregivers perceive PSAPs in terms of its function, indications, and efficacy.

Thus, in this hospital-based, multi-center, cross-sectional survey study, we investigated the current perception of PSAPs in five aspects of knowledge, needs, cost, expectation and information, and analyzed influencing factors among outpatient visitors. We also conducted an opinion survey of hearing experts to investigate the perceptions of PSAPs among hospital visitors and the professionals they meet.

## Materials and methods

### Participants

**Fig 1:**
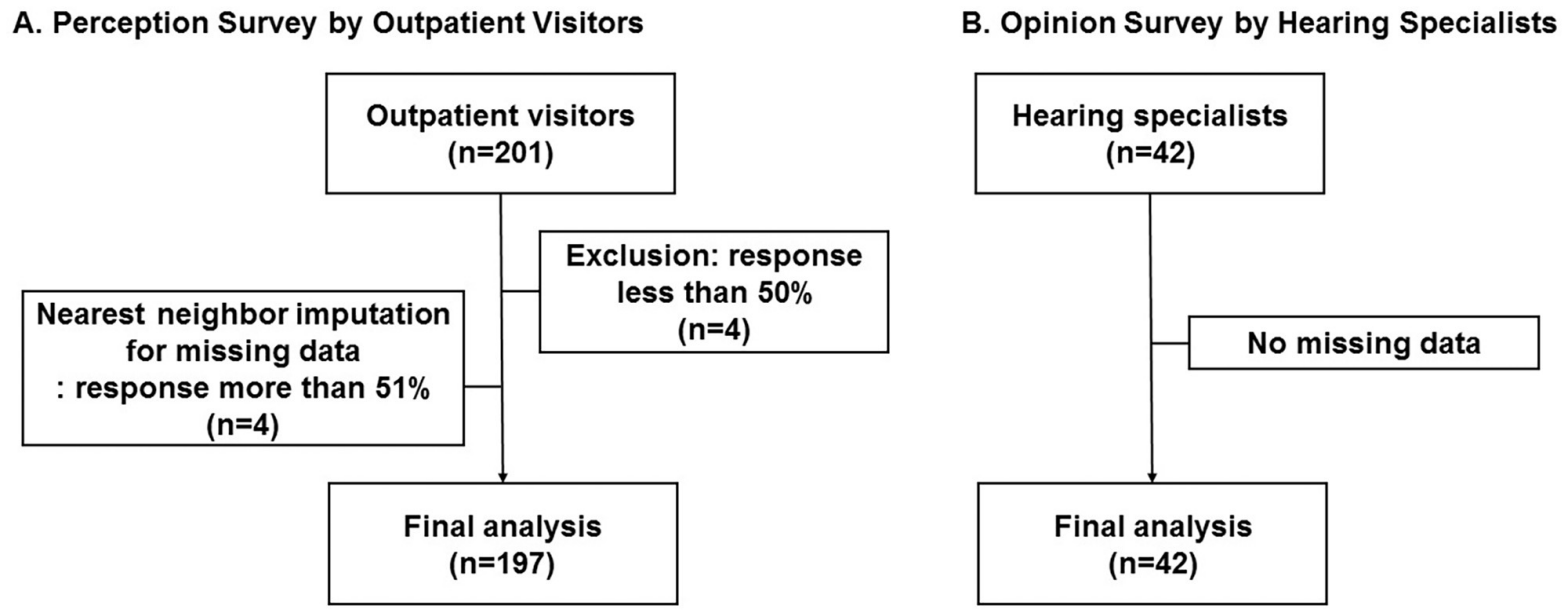
A flow chart of the methods. (A) Perception survey was conducted on 201 outpatient visitors. Outpatient visitors who responded less than 50% (n = 4) were excluded. 4 outpatient visitors responded more than 51% but did not finish the survey, and the missing data of these outpatient visitors was handled with nearest neighbor imputation method. Total of 197 outpatient visitors were included and analyzed for the perception survey. (B) Opinion survey was conducted on 42 hearing specialists, and there was no missing data.

This study was designed as a multi-center, cross-sectional survey study consisting of two groups of respondents. First, a perception survey was conducted on outpatient visitors. Outpatient visitors consist of any patient or caregiver who visited an otorhinolaryngology outpatient clinic in 5 general hospitals. Participants who had been informed of and agreed to participate in this study filled out a questionnaire under the guidance of a Clinical research coordinator (CRC), who is a nurse trained for this purpose. Second, an opinion survey about the expected price of PSAPs was conducted among hearing specialists by e-mail (Fig 1).

A total of 197 outpatient visitors who responded the perception survey to more than 50% (10 questions) were included, and 42 hearing specialists who responded to the opinion survey were included.

### Questionnaire for the perception survey

There is no standardized questionnaire to assess perception of HAs, so the survey by Park et al. was modified for use in this study [18]. The questionnaire by Park et al., which consisted of 19 questions with an appropriate level of reliability (Cronbach’s alpha of 0.757), was modified to use for this exploratory study. One question (“I know that different types of hearing aids can be worn depending on the degree of hearing loss.”) that was irrelevant to PSAPs was excluded, and “hearing aids” was replaced with “personal sound amplification products.” The final questionnaire included 18 questions. The questionnaire was written in the Korean language, and translated version of the questionnaire is attached in S1 Appendix. CRC helped the respondents throughout the survey to prevent any confusion.

The questionnaire included three components: 1) socio-demographic characteristics including age, gender, residence, educational background, economic status, and occupation; 2) clinical characteristics including subjective hearing loss, presence of tinnitus, previous experience with assistive devices by the respondent or his/her family members, respondent willingness to use assistive devices, and expected cost of the PSAPs; and 3) perception status. In the clinical characteristics section, the respondents with hearing loss or tinnitus were asked to evaluate the severity of their symptoms using a visual analogue scale (VAS). In the perception status section, the respondents were asked to respond to 18 questions in five categories on a scale from 1 (strongly disagree) to 5 (strongly agree). The questions were grouped into five categories with similar objectives, which were reviewed by the hearing specialists. Questions 1–4, which were grouped in the knowledge category, evaluated whether the respondents were aware of PSAPs as hearing rehabilitation options and the differences between PSAPs and conventional HAs. Questions 5–6 were grouped in the needs category because they were designed to assess whether the respondents thought assistive hearing devices (AHDs) were necessary for individuals with subjective hearing loss. Questions 7–8, which evaluated the influence of price on decision to purchase, were grouped in the cost category. Questions 9–13 evaluated the respondents’ expectations for the PSAPs and were grouped in the expectation category. Questions 14–18 were grouped in the information category because they were designed to assess whether participants had accurate directions for use of PSAPs. To minimize any bias that could arise from answering the questionnaire, misconception questions were included and were scored in reversal. (Question 3, 4, 10, 14, 15 and 17)

### Questionnaire for the opinion survey

The opinion survey for hearing specialists contained questions asking the expected price of PSAPs. The questionnaire was written in the Korean language, and translated version of the questionnaire is attached in S2 Appendix. Korean Won (KRW) was used as the standard currency in the questionnaire and was converted into United States dollars (USD) in this report according to the average exchange rate in 2020 (1 USD = 1,111 KRW). 42 hearing rehabilitation specialists participated in the opinion survey.

### Statistical analysis

Categorical variables were age, gender, education background, and economic status data. Reference variables were 20–39 years for age, male for gender, middle school graduate for educational background, and very high level for economic status. Continuous variables were VAS score of hearing loss and VAS score of tinnitus. Robust linear models with perception level and expected annual subscription rate as the response variables were applied. Robust variance estimation was used for standard errors and confidence intervals. In robust models, age, gender, educational background, economic status, VAS score of hearing loss, and VAS score of tinnitus were used as explanatory variables. Bonferroni-corrected *P*-values < .05 were considered statistically significant. All statistical analyses were performed using R version 3.6.0.

### Ethical consideration

This study was carried out in accordance with the Declaration of Helsinki on biomedical research for human subjects, and the study protocol was approved by the institutional review board of each participating hospital (Seoul St. Mary’s Hospital, KC20QIDI0526; Chungnam National University Hospital, 2020-06-092; Korea University Hospital, 2020GR0020; Samsung Medical Center, 2020-05-056; Seoul National University Hospital, D-2003-028-1109).

## Results

A total of 201 outpatient visitors agreed to participate in the study. Four participants who did not respond to more than 50% (10 questions) of the survey were excluded, and the survey results of 197 respondents were used for analysis. Clinical characteristics of the study population are described in Table 1.

**Table 1:** Participant characteristics.

Participant Characteristics	PSAP Perception Survey
	(n = 197)
Mean age (yr)	53.65 (14.97)
Age group	
20–39 years old	41 (20.81%)
40–59 years old	75 (38.07%)
≥ 60 years old	81 (41.11%)
Sex (male: female)	69:128 (35.03%: 64.98%)
Education level	34:56:107 (17.26%:28.43%:54.32%)
(Junior high graduate or less: high school graduate:college graduate or higher)	
Self-estimated economic status	10:23:105:45:14
(low:middle-low:middle:high-middle:high)	(5.08%:11.65%:53.30%:22.84%:7.11%)
Residence area (urban:suburban:rural area)	126:38:33 (63.96%: 19.29%: 16.75%)
Subjective hearing loss (Y:N)	116: 81 (58.88%: 41.12%)
If hearing loss ‘Y,’	3.28 (3.34)
VAS score (1–10) ^d^	
Tinnitus (Y:N)	93: 104 (47.21%: 52.79%)
If tinnitus ‘Y,’ VAS score (1–10)^e^	2.75 (3.37)

Numbers in brackets are standard deviations or percentages. SD, standard deviation; Y, yes; N, no; VAS, visual analogue scale.

^d^ VAS 1 = very minimal problem, VAS 10 = very serious problem. VAS 0 was considered ‘no subjective hearing loss.’

^e^ VAS 1 = very minimal problem, VAS 10 = very serious problem. VAS 0 was considered ‘no subjective tinnitus.’

Forty-two hearing specialists responded to the opinion survey, including 29 otologists (69.0%) and 13 audiologists (31%). Mean duration of audiologic occupation was 11.83 years (95% confidence interval, CI, 9.44–14.22). Seventeen specialists had a Bachelor’s degree (40.5%), 14 specialists had a Master’s degree (33.3%), and 11 specialists had a Doctoral degree (26.2%).

### Overall perception of PSAPs

Among the 197 outpatient visitors who responded to more than 50% of the survey questions, 50 (25.4%) had considered using AHDs, and 23 (11.7%) recognized the difference between PSAPs and HAs. Five respondents (2.5%) had used PSAPs, and only 2 respondents (1.0%) were using a PSAP at the moment. Among the 192 respondents who had never used PSAPs, 21 (10.94%) replied that they would consider using PSAPs in the future.

Of 197 respondents, 115 respondents had subjective hearing loss. Of 115 respondents with subjective hearing loss, 48 (41.7%) had considered assistive hearing devices, 14 (12.2%) recognized the difference between PSAPs and HAs, and 4 (3.5%) had experienced PSAPs. 111 respondents had subjective hearing loss, but had no experience in PSAPs; of these respondents, 15 (13.5%) responded that they would consider using PSAPs in the future.

**Fig 2:**
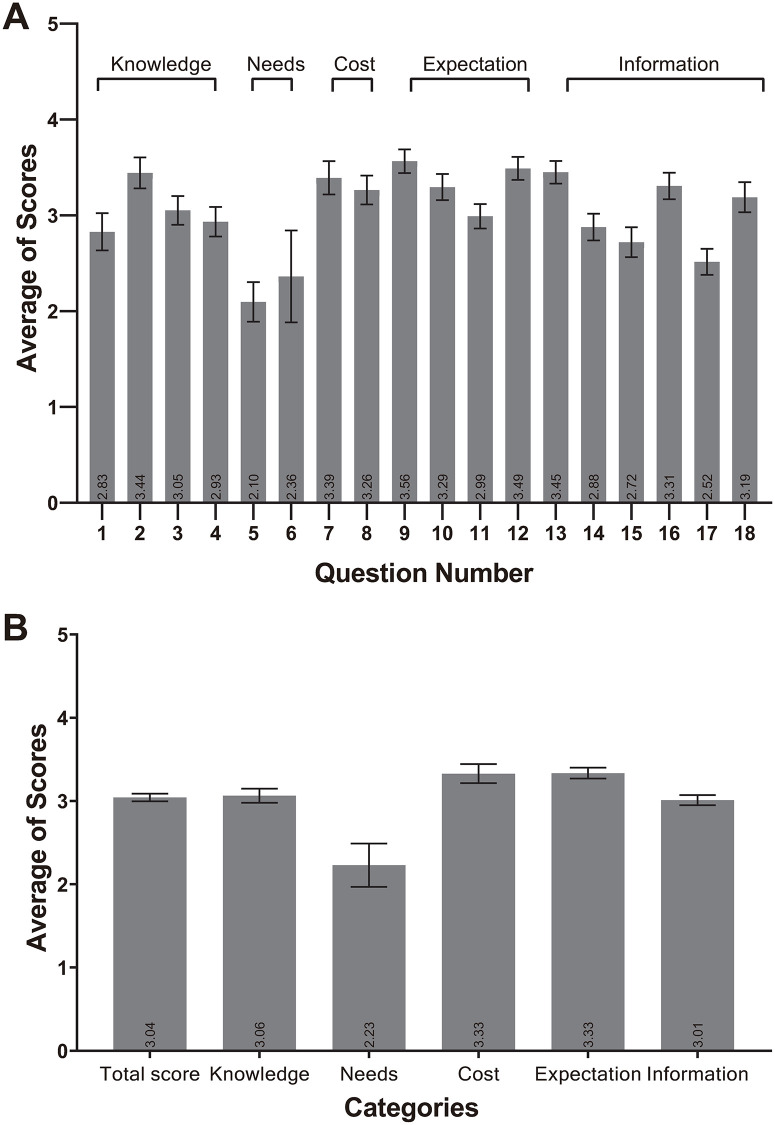
Average scores of the perception survey. (A) Average scores of 18 questions (rated by question). (B) Average scores of five categories (rated by category). Error bars indicate confidence interval (CI).

Overall perception score of 18 questions was 3.04 (95% CI, 3.00–3.09). When analyzed in 5 categories, cost and expectation categories showed the highest scores of 3.33 (95% CI, 3.21–3.44) and 3.33 (95% CI, 3.27–3.40), respectively, and the needs category showed the lowest score of 2.23 (95% CI, 1.97–2.49) (Fig 2).

### Factors affecting perception scores

#### Sociodemographic factors

To investigate whether perception of PSAPs differs by age, we divided the respondents into age groups of 20–39 years, 40–59 years, and 60 years and older (≥ 60-year-old). No statistically significant difference in perception of PSAPs was found between the 20-39-year-old group and the 40-59-year-old group. The ≥ 60-year-old group showed statistically significantly higher value on the total perception scores on PSAPs, when compared to the 20-39-year-old group (*P* = 0.028); of 5 categories, cost (*P* = 0.020) and information (*P* = 0.020) categories showed a significantly higher scores than the 20-39-year-old group (Table 2). No significant differences in perception of PSAPs according to sex, level of education, socioeconomic status, or area of residence were found.

**Table 2:** Relationships between age and perception scores on personal sound amplification products. Reference age group was the 20-39-years-old group.

Response	Coefficient	Standard Error	CI (Left)	CI (Right)	*P*-value	Adjusted *P*-value
**40-59-years-old group**						
Knowledge	0.008	0.136	-0.259	0.275	0.952	1.000
Needs	0.048	0.228	-0.399	0.495	0.832	1.000
Cost	0.250	0.176	-0.095	0.595	0.161	0.805
Expectation	0.156	0.132	-0.103	0.415	0.240	1.000
Information	0.148	0.101	-0.050	0.346	0.149	0.745
Total	0.096	0.092	-0.085	0.276	0.303	
**≥ 60-years-old group**						
Knowledge	0.056	0.165	-0.267	0.379	0.736	1.000
Needs	0.246	0.276	-0.295	0.787	0.373	1.000
Cost	0.623	0.214	0.204	1.042	0.004	**0.020** *
Expectation	0.298	0.160	-0.016	0.612	0.067	0.335
Information	0.360	0.123	0.119	0.601	0.004	**0.020** *
Total	0.248	0.112	0.029	0.467	**0.028** *	

CI: confidence interval.

* *P* < 0.05,

** *P* < 0.01,

*** *P* < 0.001.

#### Audiologic factors

Perception of PSAPs according to subjective hearing loss showed significant difference only in questions 5–6 in the needs category; individuals with subjective hearing loss showed significantly higher score on questions 5–6 in the needs category, when compared to the individuals without subjective hearing loss (*P* < 0.001). Individuals with subjective hearing loss did not show significant difference in total score when compared to the individuals without subjective hearing loss. However, individuals with higher VAS score for hearing loss showed statistically significantly higher value on total score than those with lower VAS score for hearing loss (Table 3). Severity of tinnitus and perception of PSAPs showed no significant correlation.

**Table 3:** Relationships between subjective hearing loss and perception scores regarding personal sound amplification products.

Response	Coefficient	Standard Error	CI (Left)	CI (Right)	*P-value*	Adjusted P-value
**Subjective hearing loss (yes or no)**						
Knowledge	0.005	0.018	-0.030	0.040	0.789	1.000
Needs	0.237	0.031	0.176	0.298	<0.001	**<0.001** ***
Cost	0.036	0.024	-0.011	0.083	0.134	0.670
Expectation	0.010	0.018	-0.025	0.045	0.590	1.000
Information	0.003	0.014	-0.024	0.030	0.827	1.000
Total	0.036	0.021	-0.005	0.077	0.093	
**Subjective hearing loss (VAS score)**						
Knowledge	0.008	0.030	-0.051	0.067	0.798	1.000
Needs	0.306	0.066	0.177	0.435	<0.001	**<0.001** ***
Cost	0.044	0.037	-0.029	0.117	0.227	1.000
Expectation	-0.011	0.028	-0.066	0.044	0.707	1.000
Information	-0.014	0.024	-0.061	0.033	0.560	1.000
Total	0.039	0.012	0.015	0.064	**0.002** **	

CI: confidence interval.

* *P* < 0.05,

** *P* < 0.01,

*** *P* < 0.001.

### Expected price of PSAPs and influencing factors

The expected price of PSAPs by outpatient visitors was 933.9 USD (95% CI, 811.9–1056.0) per ear. The expected price of PSAPs showed significant difference by age group. The expected price by self-estimated economic status was statistically significantly different in low and very low group; low (*P* = 0.033) and very low group (*P* = 0.020) showed significantly lower expected price of PSAPs when compared to very high group. The respondents with subjective hearing loss showed significantly higher expected price of PSAPs than the respondents without subjective hearing loss (*P = 0*.*048)*. Other factors such as gender, level of education, and tinnitus VAS scores did not show significant difference in expected price of PSAPs (Table 4).

**Table 4:** Factors affecting the expected price of personal sound amplification products.

Variable	Coefficient	Standard Error	CI (Left)	CI (Right)	*P-value*
**Age (reference: 20–39 years)**					
40–59 years	-29.798	13.985	-57.208	-2.388	**0.035** *
≥60 years	-31.272	16.927	-64.448	1.904	0.069
**Sex (reference: female)**	-9.259	11.606	-32.006	13.488	0.430
**Education level (reference: Junior high school graduate or less)**					
High school graduate	-25.415	17.172	-59.072	8.242	0.139
University graduate	-33.255	18.792	-70.087	3.577	0.082
**Self-estimated economic status (reference: very high)**					
High	-31.804	26.085	-82.93	19.322	0.238
Middle	-35.070	23.415	-80.963	10.823	0.149
Low	-54.984	25.108	-104.195	-5.773	**0.033** *
Very low	-72.571	30.327	-132.011	-13.131	**0.020** *
Subjective hearing loss (VAS score)	3.822	1.858	0.180	7.464	**0.048** *
Tinnitus (VAS score)	-1.655	1.692	-4.971	1.661	0.340

CI: confidence interval. VAS, visual analogue scale.

* *P* < 0.05,

** *P* < 0.01,

*** *P* < 0.001.

**Fig 3:**
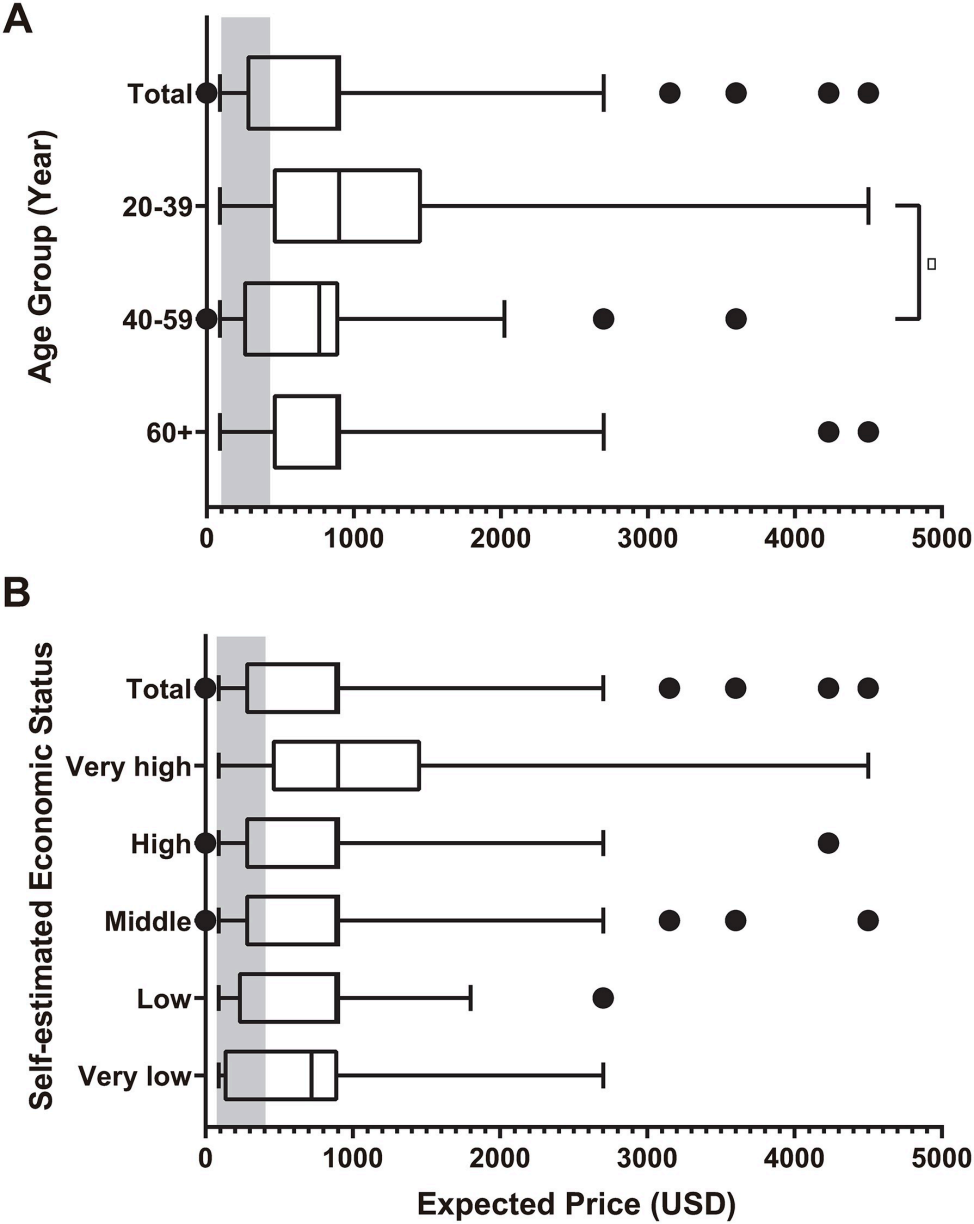
Expected price of personal sound amplification products (PSAPs). (A) Expected PSAP price according to age groups (20–39, 40–59, ≥ 60-year-old). Mean expected price of the 20-39-year-old group (1123.2 USD, 95% CI, 772.1–1474.3) was significantly higher than that of the 40-59-year-old group (745.5 USD, 95% CI, 590.4–900.5, *P* < 0.026). (B) Expected PSAP price according to self-estimated economic status. Mean expected prices of the low group (724.3 USD, 95% CI, 797.5–1141.1) and the very low group (742.6 USD, 95% CI, 344.3–1140.8) were lower than that of the very high group (1201.4 USD, 95% CI, 869.4–1533.4). A box and whisker plot shows summary of a set of data: Maximum, 75 percentile, median, 25 percentile and minimum. Grey area indicates real market price of PSAPs. USD, United States dollar.

Mean expected price in the 20-39-year-old group was 1123.2 USD (95% CI, 772.1–1474.3), 745.5 USD (95% CI, 590.4–900.5) in 40-59-year-old group, and 1014.2 USD (95% CI, 819.7–1208.7) in the ≥ 60-year-old group. Individuals in the 20-39-year old group showed statistically significantly higher willingness to pay higher prices compared to the 40-59-year old group (P = 0.026) (Fig 3A).

In terms of self-estimated economic status, individuals in the low group (724.3 USD, 95% CI, 797.5–1141.1, *P* = 0.033) and in the very low group (742.6 USD, 95% CI, 344.3–1140.8, *P = 0*.*048*) showed statistically significantly lower value on mean expected price, when compared to the very high group (1201.4 USD, 95% CI, 869.4–1533.4). In other words, low and very low groups showed lower willingness to pay than the very high group (Fig 3B).

**Fig 4:**
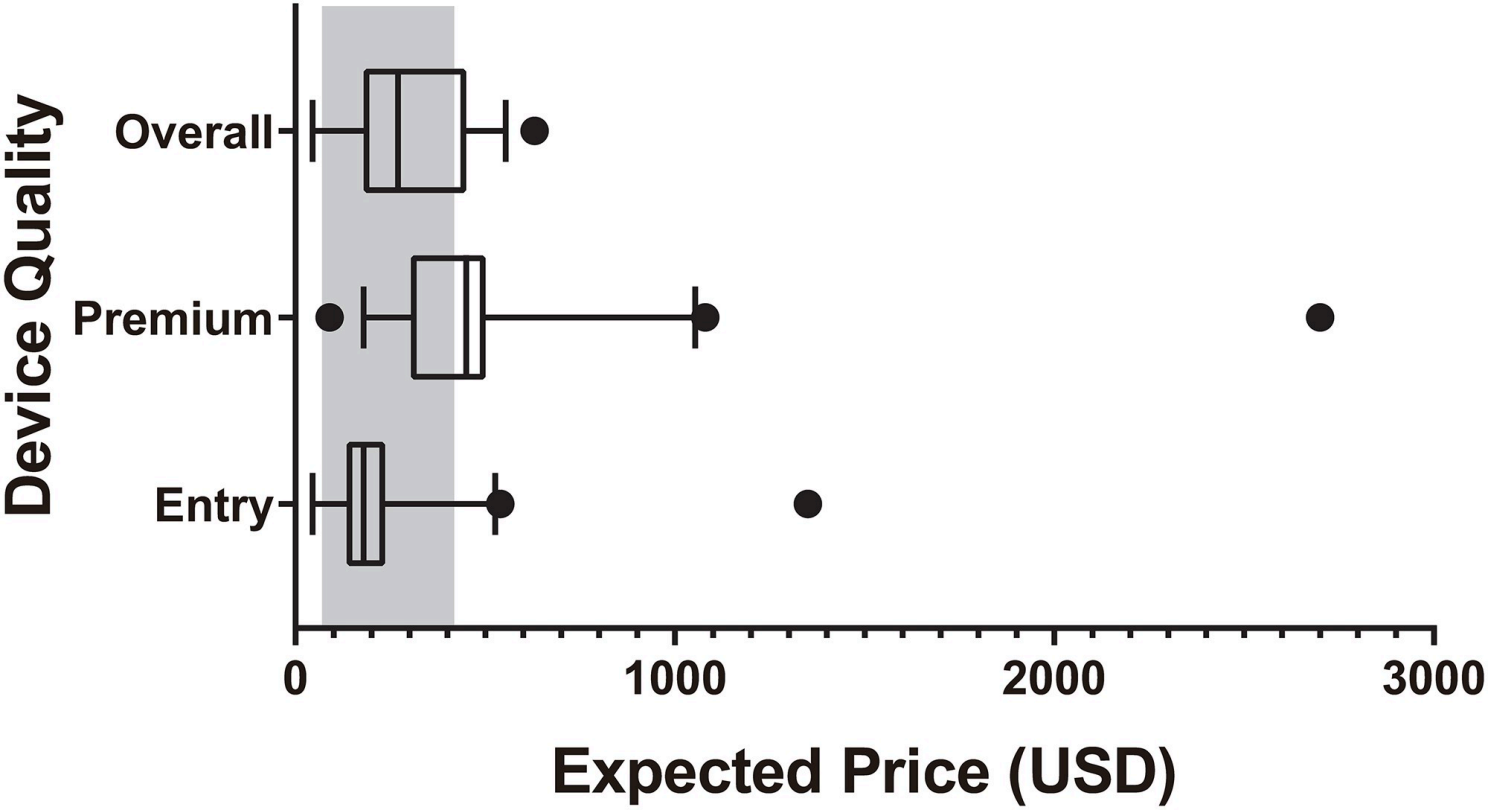
Experts opinion on the price of personal sound amplification products (PSAPs). A box and whisker plot shows summary of a set of data: Maximum, 75 percentile, median, 25 percentile and minimum. Grey area indicates real market price of PSAPs. US $, United States dollar.

Mean expected price of PSAPs by hearing specialists was 291.3 USD (95% CI, 238.2–344.3) per ear; mean expected price of premium PSAPs was 487.6 USD (95% CI, 365.4–609.8), and that of entry PSAPs was 224.9 USD (95% CI, 161.9–287.9) (Fig 4). In addition, the selling price of PSAPs in Republic of Korea was investigated by web-based searching (S2 Appendix) and the current price of PSAPs was 70–630 USD, which is indicated as a grey zone in the figure.

## Discussion

In this study, overall perception of PSAPs was investigated in 197 outpatient visitors who visited an otorhinolaryngology outpatient clinic in five general hospitals. Of 197 outpatient visitors, only 23 (11.7%) had information about PSAPs, and among 116 individuals with subjective hearing loss, only 14 (12.2%) recognized the difference between PSAPs and HAs. Thus, regardless of presence of hearing loss, overall perception of PSAPs is poor in Republic of Korea. Subjective hearing loss and tinnitus did not show significant correlation to the knowledge category score in the perception survey, indicating that even individuals with hearing difficulty or tinnitus do not have enough information about PSAPs.

Among individuals with subjective hearing loss, only 5 (3.5%) had experience with PSAPs. This is much lower than the PSAP adoption rate of 9.6% in individuals with hearing difficulties in the United States [13]. In this study, since we relied on the survey responses by the respondents, it was not possible to check if the 5 respondents who responded to have experience with PSAPs actually used them; the respondents could have mistakenly considered other types of AHDs as PSAPs. In fact, among 5 individuals who responded that they had previous experience using PSAPs, only 1 individual (20%) could correctly distinguish between PSAPs and HAs. MarkeTrak 10 also reported that more than half of PSAP users do not know which device they are using or mistakenly consider PSAPs as Has [19]. Thus, the actual adoption rate of PSAPs among individuals with hearing difficulty could be lower than in our study.

When the perception of PSAPs of all respondents was analyzed in 5 categories, the score of the needs category was the lowest, and this could be because the perception survey included both individuals with normal hearing and individuals with subjective hearing loss. However, individuals with subjective hearing loss showed significantly higher score in the needs category than the individuals without subjective hearing loss. In other words, individuals with subjective hearing loss showed greater necessity for AHDs than individuals without subjective hearing loss. MarkeTrak VIII and EuroTrak data reported that hearing loss plays the most important role in HA adoption [20, 21]. Although we did not investigate the correlation between hearing loss and PSAP adoption rate, we have found significant correlation between hearing loss and the need for AHDs. In addition, individuals with subjective hearing loss showed statistically significantly higher expected price of PSAPs than the individuals without subjective hearing loss. Individuals with subjective hearing loss showed greater needs for hearing rehabilitation with AHDs, which led to willingness to pay higher price for PSAPs.

No statistically significant correlation was shown between perception score and tinnitus in any category. This result is similar to the MarkeTrak VIII and EuroTrak data, which showed that tinnitus is not an important factor for HA adoption. Thus, hearing loss is more critical than tinnitus in selecting AHDs [20, 21].

The ≥ 60-years-old group showed significantly higher scores on information and cost categories, when compared to the 20-39-year-old group; in other words, the ≥ 60-years-old group has more information on the use of PSAPs and is more greatly influenced by the price on making decision to purchase PSAPs. The 20-39-year-old group showed highest mean expected price of PSAPs. This could be explained by low score on information of the 20-39-year-old group, when compared to the ≥ 60-years-old group; the 20-39-year-old group did not have enough information on PSAPs to accurately predict the price. In addition, those with subjective hearing loss in the 20-39-year-old group (26.3%) could have higher will for hearing rehabilitation because they are young, and this could have led to willingness to pay higher prices for PSAPs.

In terms of self-estimated economic status, the low-to-very low group showed statistically significantly lower value on mean expected price than the very high group; the middle and high groups showed no significant difference in mean expected price. Previous studies have reported that cost is not a primary barrier to adoption of AHDs and that other factors such as social stigma, perception of hearing loss, and self-efficacy are equally important [7, 22]. Our data supports this finding, showing that the mean expected price does not show significant correlation with the self-estimated economic status, except for the low-to-very low group. Cost is one of the factors that decides adoption of AHDs [3], and in this study, we found that cost is especially important in individuals with low-to-very low economic status.

In addition, overall expected price of the outpatient visitors was 933.9 USD (95% CI, 811.9–1056.0) per side, which was much higher than the real market price of 20–400 USD in the United States and 70–630 USD in Republic of Korea [12]. Large discrepancy between the expected and the real market price could be due to poor perception of PSAPs among the respondents. As mentioned earlier, only 11.3% had information about PSAPs, and among individuals with subjective hearing loss, only 12.2% could differentiate between PSAPs and HAs. The respondents could have mistakenly considered PSAPs as similar devices to HAs, thus expecting PSAPs to price as much as HAs.

On the other hand, the expected price from the expert opinion survey was within a range of the real market price. In other words, current price policy of PSAPs is provider-oriented, rather than customer-oriented, and possible consumers of PSAPs are willing to pay more for better performance. Therefore, it is necessary for the hearing specialists to educate the general population, especially those in needs, on the real market price of PSAPs to encourage PSAP adoption.

One of the strengths of this study is that this is the first perception survey and analysis focused on PSAPs to our knowledge. MarkeTrak 10 investigated PSAPs in addition to HAs, but there was no scientific analysis on PSAPs [19]. In particular, this is the first study on PSAPs in Republic of Korea. In the past, there was a survey study of HAs on the basis of MarkeTrak, but it was limited to investigating satisfaction in Has [23]. A previous perception survey was limited to HAs, targeting the general public only in Busan [24]. Another strength of this study is that this study was conducted as a multi-center study, comprising general hospitals in various regions and minimizing regional bias. The rate of urban to suburban area residents in this study was 83.3%, which was lower than that of Republic of Korea in 2019, which was 91.1% [25].

One of the limitations of this study is that we could only use a modified survey questionnaire from a previous study conducted in Republic of Korea due to the lack of an official survey form regarding PSAPs. This previous study was conducted by Park et al., and they used a self-verified questionnaire. The questionnaire from the previous study was used to study HAs, so it was modified with regards to PSAPs [24]. Therefore, it is necessary to develop an official questionnaire with consensus to continue perception survey of AHDs. Another limitation of this study is that both the patient and caregiver from the same family could have answered the questionnaire, which could have resulted in replication of the economic status information. In this study, we have conducted survey on any outpatient visitor sitting in the waiting room of the otorhinolaryngology outpatient clinic. We have not differentiated patients from caregivers and did not ask if the respondent was a patient or a caregiver. Thus, both the patient and caregiver from the same family could have been included in this study. Respondents from the same family would show similar economic status, so there could be a replication of economic status information. Further cross-sectional survey study with more randomized population could improve this limitation.

Due to multi-factorial barriers, the role of conventional HAs still has limitations in reaching individuals with hearing loss [7, 14, 22]. Alternative AHDs, such as PSAPs, can be good options for hearing rehabilitation before the patients recognize subjective hearing loss and adopts conventional Has [26]. The global market of hearing amplifiers is progressively expanding with a compound annual growth rate (CAGR) of 6.6% (from USD 62.6 million in 2019 to USD 105.2 million in 2027). The Asia-Pacific market is small, but it has a greater growth potential compared to those of North America or Europe [27]. In addition, clinical performance of PSAPs is improving, and the effectiveness of PSAPs has been validated; we expect their role to lower barriers in individuals with mild to moderate hearing difficulties to expand further [15, 16, 28, 29]. However, we found in this study that the current perception of PSAPs in South Korea is very poor, and the actual use of PSAPs were much lower when compared to that of the United States. Therefore, the hearing specialists should know the characteristics, strengths and weaknesses, and the real market price of PSAPs and try to deliver accurate information to the general public, especially the individuals with subjective hearing loss, to increase PSAP adoption in South Korea.

In this study, the perception rate of PSAPs was very poor, and age and subjective hearing loss were closely related to perception. Expected price by hearing experts was similar to the real market price, but the otorhinolaryngology outpatient visitors were willing to pay more. Therefore, hearing specialists need to provide accurate information to improve perception of PSAPs and ultimately encourage the patients in need to adopt PSAPs.

## Supporting information

S1 Appendix Awareness survey. (DOCX)

S2 Appendix Specialist survey. (DOCX)

S3 Appendix PSAPs in Republic of Korea. (XLSX)
