# Supplementary material for: Perception and expectations of personal sound amplification products in Korea: A hospital-based, multi-center, cross-sectional survey
Source: PLoS One. 2022 May 26;17(5):e0269123. doi: 10.1371/journal.pone.0269123 (PMC9135199; doi:10.1371/journal.pone.0269123)
Supplement: S1 Appendix — (DOCX) [file pone.0269123.s001.docx]

Personal Sound Amplification Products (PSAPs): Awareness Survey

Date: MM/DD/YYYY

⋇ Please indicate ◯ or ⩗ in the following questions.

1. Date of birth; month and year only.

(MM/YYYY)

2. What is your gender?

① Male ② Female

3. Where do you currently live? Please indicate city, county, or district level. (Example: Seocho-gu, Seoul)

( )

4. Are you living with your children?

① Yes ② No

5. Please indicate your education level.

① Junior high school graduate or less ② High school graduate ③ College graduate or higher

6. What would your rank your economic status as?

① High ② High-Middle ③ Middle ④ Middle-Low ⑤ Low

7. Please indicate your current job. If you are currently unemployed, please indicate your past job.

(1) Executive (2) Experts/Professionals (3) Administrative

(4) Customer Service Representative (5) Salesperson

(6) Agricultural, Forestry, Fishery-related (7) Technician and Related Functional

(8) Machinist or Assembly-related (9) Simple Labor (10) Soldier

(11) Student (12) Housewife (13) Unemployed (14) Other: ( )

8. Have you ever been diagnosed with any of the following conditions?

(1) Diabetes: Yes / No

(2) Hypertension: Yes / No

(3) Other: ( )

9. Do you think you have a problem with hearing?

① Yes ② No

10. (If you think you have a problem with hearing) Please mark it how serious the problem is.

│──│──│──│──│──│──│──│──│──│──│

0 1 2 3 4 5 6 7 8 9 10

(No problem at all) (Serious problem)

11. Do you have tinnitus often?

① Yes ② No

12. (If you have tinnitus) Please mark it how severe it is.

│──│──│──│──│──│──│──│──│──│──│

0 1 2 3 4 5 6 7 8 9 10

(No tinnitus) (Very severe tinnitus)

13. Have you ever considered or been recommended to use assistive devices for hearing, such as hearing aids or personal sound amplification products (hereinafter referred to as PSAPs)?

① Yes ② No

14. Do you know what the difference is between hearing aids and PSAPs?

① Yes ② No

15. Have you ever used PSAPs?

① Yes ② No

If yes, how long have you used them for: ( )

Are you currently using them? ( )

16. Does anyone in your family or an acquaintance use PSAPs?

① Yes ② No ③ I don’t know

17. Have you ever heard an explanation or seen an advertisement for PSAPs?

① Yes ② No

18. If you were offered a PSAPs, would you be willing to use it?

① Yes ② No ③ I don’t know

19. In order to solve the discomfort caused by hearing loss, how much are you willing to pay for PSAPs? Please indicate what you would be willing to pay for use of PSAPs for a period of 1 year.

│─────│─────│─────│─────│─────│

$0 $50 $100 $150 $200 $300

You can also write a specific price. ( )

※ Your perception of “Personal Sound Amplification Products (PSAPs)”

| Personal Sound Amplification Products (PSAPs): Awareness Survey | Strongly  Agree | Slightly  Agree | Agree | Slightly  Disagree | Strongly  Disagree |
| --- | --- | --- | --- | --- | --- |
|  | 5 | 4 | 3 | 2 | 1 |
| PSAPs are one way to make soft sound audible. | 5 | 4 | 3 | 2 | 1 |
| PSAPs are different from hearing aids. | 5 | 4 | 3 | 2 | 1 |
| PSAPs are a type of hearing aid. | 5 | 4 | 3 | 2 | 1 |
| PSAPs are an inexpensive replacement for hearing aids. | 5 | 4 | 3 | 2 | 1 |
| I have considered using hearing aids (hearing aids, sound amplifier, or PSAPs) because sounds are soft. | 5 | 4 | 3 | 2 | 1 |
| I have considered using hearing aids (hearing aids, sound amplifier, or PSAPs) because I don't understand words well even if I can hear sound. | 5 | 4 | 3 | 2 | 1 |
| Cost is the most important factor to consider when deciding whether to use PSAPs. | 5 | 4 | 3 | 2 | 1 |
| The more expensive the PSAPs, the better it allows sound to be heard. | 5 | 4 | 3 | 2 | 1 |
| Sound might be loud if using PSAPs. | 5 | 4 | 3 | 2 | 1 |
| Unwanted sounds might be amplified when using PSAPs. | 5 | 4 | 3 | 2 | 1 |
| I have normal hearing when using a PSAPs. | 5 | 4 | 3 | 2 | 1 |
| I can understand words when using a PSAPs. | 5 | 4 | 3 | 2 | 1 |
| The more consistent the use of PSAPs, the better the outcome. | 5 | 4 | 3 | 2 | 1 |
| PSAPs should be as high-powered as possible. | 5 | 4 | 3 | 2 | 1 |
| PSAPs should be used when sounds are barely heard. | 5 | 4 | 3 | 2 | 1 |
| The sooner I use PSAPs, the better the sound gets. | 5 | 4 | 3 | 2 | 1 |
| Hearing loss might be worsened by using PSAPs. | 5 | 4 | 3 | 2 | 1 |
| When using PSAPs, it is important to use it on both ears. | 5 | 4 | 3 | 2 | 1 |
