# Supplementary material for: Perception and expectations of personal sound amplification products in Korea: A hospital-based, multi-center, cross-sectional survey
Source: PLoS One. 2022 May 26;17(5):e0269123. doi: 10.1371/journal.pone.0269123 (PMC9135199; doi:10.1371/journal.pone.0269123)
Supplement: S2 Appendix — (DOCX) [file pone.0269123.s002.docx]

Survey: Hearing Specialist’s Opinion for PSAPs (Personal sound amplification products)

Date: MM/DD/YYYY

⋇ Please indicate ◯ or ⩗ in the following questions.

1. Check your current status

① Otologist

② Audiologist

2. Where are you currently working for?

① University hospital

② Private clinic

③ University (Audiology, Medical engineering, etc)

④ Company

⑤ Others

3. How long have you worked in the field of audiology or otology since graduating from college?

: ( ) years

4. What is your final degree?

① Bachelor's

② Master's

③ Doctor's

5. What is your expected retail price for premium and entry PSAPs?

- Premium PSAP ( ) Won

│─────│─────│─────│─────│─────│

$0 $50 $100 $150 $200 $300

- Entry PSAP ( ) Won

│─────│─────│─────│─────│─────│

$0 $50 $100 $150 $200 $300
